# High frequency of Enterococcus faecalis detected in urinary tract infections in male outpatients – a retrospective, multicenter analysis, Germany 2015 to 2020

**DOI:** 10.1186/s12879-023-08824-6

**Published:** 2023-11-18

**Authors:** Jonas Salm, Florian Salm, Patricia Arendarski, Tobias Siegfried Kramer

**Affiliations:** 1 Charité – Universitätsmedizin Berlin, corporate member of Freie Universität Berlin and Humboldt-Universität Zu Berlin, Berlin School of Public Health, Berlin, Germany; 2 Department of Cardiology and Angiology, University Heart Center Freiburg - Bad Krozingen, Medical Center – University of Freiburg, Faculty of Medicine, University of Freiburg, Freiburg, Germany; 3 Prevent Infect, Bad Krozingen, Germany; 4 Charité – Universitätsmedizin Berlin, corporate member of Freie Universität Berlin and Humboldt-Universität Zu Berlin, Institute for Hygiene and Environmental Medicine, Berlin, Germany; 5 LADR Laboratory Group Dr Kramer & Colleagues, Geesthacht, Germany

**Keywords:** Urinary tract Infections, Antimicrobial resistance, Germany, Outpatient, Ambulatory, Antimicrobial stewardship, Enterococcus faecalis

## Abstract

**Background:**

Urinary tract infections (UTI) in men differ relevantly to women by their pathogens. Gram-positive uropathogens play a relevant role in UTI in men. In this study, we aimed to analyze the epidemiology of *Enterococcus faecalis* in UTI in male outpatients.

**Methods:**

We conducted a retrospective observational multicenter study during 2015 to 2020 consisting of urine samples of 99,415 adult male outpatients sent from 6,749 outpatient practices from Germany. Proportions were compared using the z-Test and 95% confidence intervals were calculated using the Clopper-Pearson method.

**Results:**

*E. faecalis* is the 2^nd^ most frequent bacteria (16%) detected in suspected UTI in male outpatients. Young men are predominantly at risk (17%) for isolation of *E. faecalis* in suspected UTI. In polymicrobial infections *E. faecalis* is isolated in 47% of all suspected UTI in men. Recurrency of suspected UTI is significantly more frequent when *E. faecalis* is isolated compared to *Escherichia coli* (22% vs 26%; p < .001). Recurrency rates of *E. faecalis* associated UTI increases by age from 12% (18–29 years) to 28% ($\ge$ 70 years); p < .001. Congruently the resistance of *E. faecalis* against ciprofloxacin increases by age from 22% (18–29 years; 2019) to 37% ($\ge$ 70 years; 2019); p < .001.

**Conclusions:**

*E. faecalis* is frequently isolated in suspected UTI in male patients. Consequently, Nitrate-sticks results cannot be recommended to exclude UTI in men. The empirical use of ciprofloxacin in young adults can be reasonable. Frequent recurrences in *E. faecalis* associated suspected UTI emphasizes the importance of microbiological pathogen identification and susceptibility testing in men suffering from UTI.

**Supplementary Information:**

The online version contains supplementary material available at 10.1186/s12879-023-08824-6.

## Background

Urinary tract infections (UTI) are one of the most common infections in humans [1]. Predominantly young females are affected by UTI and gram-negative bacteria, especially *Escherichia coli*, cause 75 – 95% of these UTI [2].

National and international guidelines categorize UTI into subgroups for empirical treatment recommendations. Despite accounting for different infection-sites and complexity of UTI, gender is one of these subgroup affiliating characteristics [3, 4].

Evidence regarding UTI in men remains scarce. Recent studies reveal *E. coli* being considerably less and *Enterococcus faecalis* being considerably more frequent in men compared to women [5–7]. Gram-positive bacteria are known for their capability of causing UTI as well, whilst the pathogenicity of *E. faecalis* remains unclear [8].

*E. faecalis* is often detected in polymicrobial infections. In female patients UTI caused by gram positive bacteria typically occur in individuals who are elderly or have catheter associated UTI [8, 9]. Despite, being the second most frequent bacteria in UTI in men, and therefore marking a major difference between female and male UTI [6, 7], the epidemiology of *E. faecalis* in male patients is not yet known.

The objective of this study was to assess the epidemiology and resistance against ciprofloxacin of UTI caused by *E. faecalis* in men 2015—2020. In addition, we compared polymicrobial infections caused by *E. faecalis* by their co-infecting pathogens as well as recurrency rates between UTI caused by *E. faecalis* and *E. coli.*

## Methods

### Study design

We retrospectively analysed data derived from routine urine cultures of male outpatients from 2015 to 2020. Urine culture diagnostics and data storage were performed by medical laboratories of the private LADR Laboratory Group Dr Kramer & Colleagues. We included data from nine of their laboratories in Germany.

To ensure that the dataset only contained positive and relevant urine cultures, we extracted the data on those bacteria known to be relevant for causing UTI according to the Quality Standards for the Microbiological Diagnosis of Infectious Diseases (Mikrobiologisch-infektiologische Qualitätsstandards; MIQ) [10]. Further, we excluded all cultures which were not from midstream urine, with a bacteria count lower than 10^4^ colony-forming units per mL and, in case of polymicrobial infections, with more than two infecting bacteria. In a next step we excluded every result with a positive test of growth inhibition of Bacillus simplex. After applying these inclusion and exclusion criteria, the remaining urine culture results were defined as representing the clinical diagnosis UTI.

We generated the binary variables 'recurrent UTI' and 'polymicrobial'. According to the German guideline, every UTI in the same patient which occurred within 6 months since the last UTI was labeled as recurrent UTI [3]. Every UTI after these 6 months was defined as a new infection. We did not consider the first 7 days after the initial UTI for the definition of recurrence, to account for multiple testing of the same initial UTI. Urine culture results on the same date with different relevant pathogen isolates were labelled as polymicrobial.

### Study participants

All male outpatients ≥ 18 years with midstream urine cultures within the years 2015 to 2020 were included in the study. These samples were sent by 6,749 outpatient practices in Germany. For every study participant, we had complete urine culture results of relevant bacteria which are known to cause UTI. Since mono- and polymicrobial infections are considered in the study, there are fewer patients than pathogen isolates.

### Microbiology

Pathogen identification and antimicrobial susceptibility testing (AST) was performed with automated systems such as MALDI-TOF, Vitek2, disc diffusion and microbroth dilution. We included susceptibility testing against ciprofloxacin (CIP) from 2015 to 2019. The results were interpreted according to the European Committee on Antimicrobial Susceptibility Testing (EUCAST) [11].

### Statistical analysis

Descriptive analyses for patient characteristics are reported as means with standard deviation (SD) for continuous variables and as counts with percentages for categorical variables. Antimicrobial susceptibility of *E. faecalis* is reported as the percentage of resistant isolates among all tested isolates. We calculated 95% confidence intervals (CI) for proportions using the Clopper–Pearson method. Proportions were compared using the z-test. All statistical analyses were performed using the free software for statistical computing and graphics R (R 4.2.0; R Foundation, Vienna, Austria). The significance level was set to α = 0.05.

## Results

### Study participants

In total 99,415 male outpatients were included into the study. The mean age of the study population was 69.2 (SD: ± 15.0) years. Men with monomicrobial infections compared to polymicrobial infections are younger with 68.5 (SD: ± 15.2) to 71.8 (SD: ± 13.9) years. Suspected recurrent UTI are less frequent in monomicrobial (22.8%) compared to polymicrobial infections (29.7%) (Table1).

**Table 1:** Studypopulation stratified by polymicrobial infections

	Overall **(*N* = 99,415)**	Monomicrobial **(*N* = 77,864)**	Polymicrobial^a^ **(*N* = 21,551)**
**Age in years**			
Mean (SD)	69.19 (14.96)	68.46 (15.15)	71.84 (13.91)
**Age in groups**			
18–29 Years	2,220 (2.2%)	1,897 (2.4%)	323 (1.5%)
30–39 Years	3,078 (3.1%)	2,609 (3.4%)	469 (2.2%)
40–49 Years	5,253 (5.3%)	4,417 (5.7%)	836 (3.9%)
50–59 Years	12,310 (12.4%)	10,224 (13.1%)	2,086 (9.7%)
60–69 Years	19,470 (19.6%)	15,693 (20.2%)	3,777 (17.5%)
70–79 Years	29,576 (29.8%)	22,888 (29.4%)	6,688 (31.0%)
80–89 Years	24,993 (25.1%)	18,341 (23.6%)	6,652 (30.9%)
≥ 90 Years	2,515 (2.5%)	1,795 (2.3%)	720 (3.3%)
**Culture**			
Recurrent UTIs^b^	24,178 (24.3%)	17,771 (22.8%)	6,407 (29.7%)
**Region**			
North	36,607 (36.8%)	28,167 (36.2%)	8,440 (39.2%)
East	12,367 (12.4%)	10,378 (13.3%)	1,989 (9.2%)
South	12,784 (12.9%)	11,241 (14.4%)	1,543 (7.2%)
West	37,657 (37.9%)	28,078 (36.1%)	9,579 (44.4%)

^a^Polymicrobial: More than one isolated pathogen per culture

^b^Recurrent UTIs: More than one urinary tract infection in the last 6 month

### Pathogen distribution

*E. faecalis* is the second most frequent bacteria in suspected UTI in male outpatients (16.1%) (See Additional file 1). Stratification by age reveals a frequency peak in men aged 18 to 29 years (16.8%). In monomicrobial infections *E. faecalis* still is the second most frequent bacteria in suspected UTI (12.0%), with men 18 to 29 years being predominantly at risk (13.9%) (See Additional file 1).

**Fig. 1:**
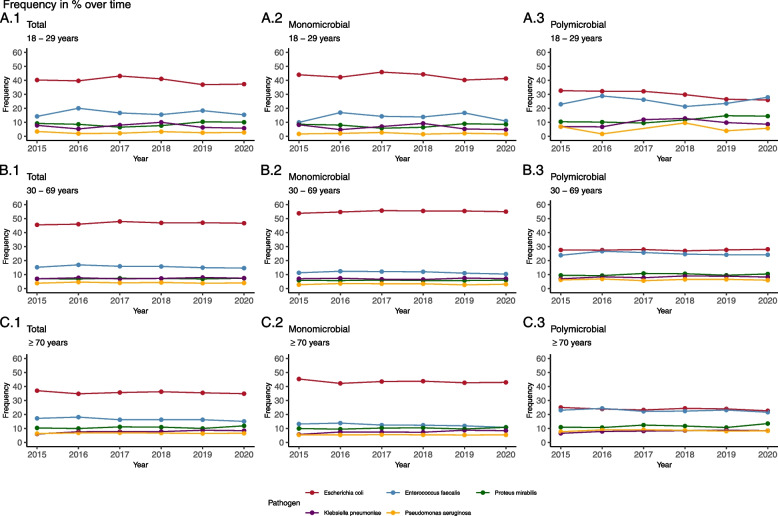
Legend: Frequency of bacteria detected in urine samples from male outpatients in percent over time. Stratified by age group, stratified and polymicrobial infections. Bacteria detected are shown in different colours

Further the prominent role of *E. faecalis* as second most frequent bacteria in suspected UTI in male outpatients, independently of stratification, is constant over time (Fig. 1).

### Polymicrobial infections

*E. coli* and *E. faecalis* are the dominant co-infecting pathogens in total. They are involved in 48.4% and 46.8% of all polymicrobial infections, respectively (Table 2).

**Table 2:** Involvement of pathogens as co-infecting pathogens in polymicrobial UTI in male outpatients, Germany 2015—2020 (*n* = 43,097) -

Pathogen (*N* = 43,097)	*n*	Frequency	95% CI
*E. coli*	20,862	48.4	47.9; 48.9
*E. faecalis*	20,174	46.8	46.3; 47.3
*P. mirabilis*	9,506	22.1	21.7; 22.5
*K. pneumoniae*	6,841	15.9	15.5; 16.2
*P. aeruginosa*	6,536	15.2	14.8; 15.5

Frequency measured as counts of certain pathogens involved into polymicrobial infections as co-infecting pathogen

Co-infecting pathogens alongside *E. faecalis* in polymicrobial infections are predominantly gram-negative bacteria with the top three consisting of: *E. coli* (42.9%), *Proteus mirabilis* (11.2%) and *Pseudomonas aeruginosa* (10.1%) (Table 3).

**Table 3:** Polymicrobial UTI: Co-infecting pathogens alongside *Enterococcus faecalis* in male outpatients, Germany 2015 – 2020 (*n* = 20,174)

	**n**	**Proportion**	**95% CI**
**Pathogen (*N* = 20,174)**			
*Escherichia coli*	8,658	42.9	42.2; 43.6
*Proteus mirabilis*	2,266	11.2	10.8; 11.7
*Pseudomonas aeruginosa*	2,042	10.1	9.7; 10.5
*Klebsiella pneumoniae*	1,726	8.6	8.2; 8.9
*Citrobacter spp.*	1,258	6.2	5.9; 6.6
*Klebsiella spp.*	1,138	5.6	5.3; 6
*Enterobacter spp.*	860	4.3	4; 4.6
*Staphylococcus aureus*	788	3.9	3.6; 4.2
*Morganella spp.*	662	3.3	3; 3.5
Others	776	3.9	-

95% CI: 95% confidence interval

### Recurrency rates

**Fig. 2:**
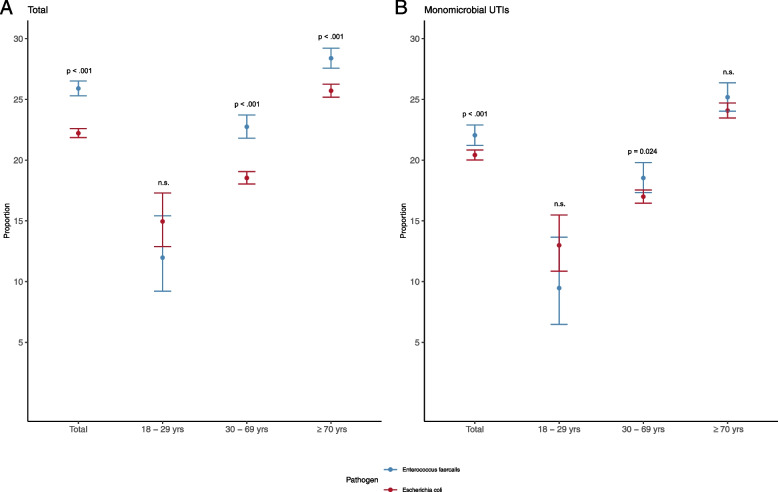
Legend: Frequencies of *Enterococcus faecalis* and *Escherichia coli* detected in recurrent urinary tract infections in male outpatients. Stratification was performed by age in groups and by monomicrobial infections. Blue indicates *E. faecalis* isolates and red indicates *E. coli* isolates. Estimates are presented with 95% confidence intervals (bars)

In total recurrency is significantly more frequent in suspected UTI if *E. faecalis* is detected (25.9%) compared to *E. coli* (22.2%; *p* < 0.001). In men aged 18 to 29 years in contrast recurrency is considerably less frequent in UTI if *E. faecalis* (12.0%) is detected compared to *E. coli* (15.0%). This pattern remained after stratification by monomicrobial infections (Fig. 2).

### Antimicrobial resistance

**Fig. 3:**
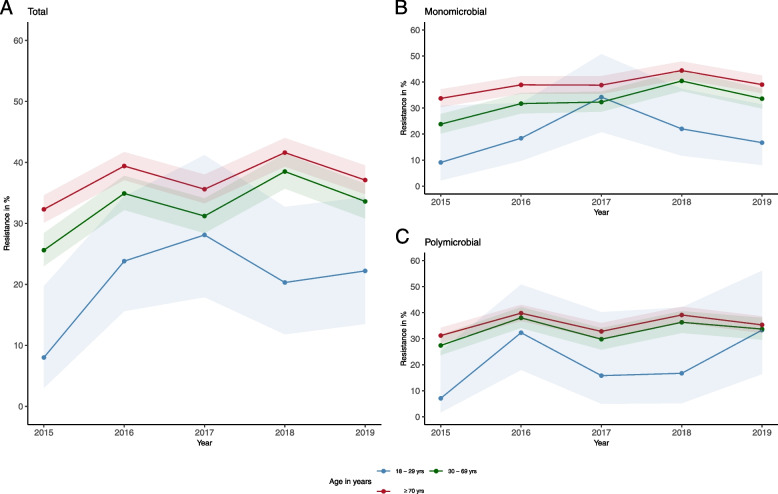
Legend: Ciprofloxacin resistance in *Enterococcus faecalis* isolates from urine specimens of male outpatients over time. Resistance is expressed as a percentage in different age groups, indicated by colours

Figure 3 illustrates antimicrobial resistance (AMR) of *E. faecalis* against ciprofloxacin (CIP) over time. In total and stratified by monomicrobial infections, CIP resistance among *E. faecalis* isolates is significantly lower over time (*p* < 0.001) in men 18 to 29 years compared to men 30 to 69 years, except for 2017 (Fig. 3). After stratification by polymicrobial infections statistically significant differences between men aged 18 to 29 years compared to men 30 to 69 years can only be detected for the years 2015, 2017 and 2018 (*p* < 0.001) (Fig. 3).

## Discussion

*E. faecalis* is the second most frequently isolated bacteria in suspected UTI in men – in both mono- as well as polymicrobial infections. Men aged 18 to 29 years are predominantly at risk for detection of *E. faecalis* in suspected UTI. In contrast, *E. faecalis* plays a minor role in UTI in women [12]. Secondly, gram-positive bacteria have a frequency peak in women 80 years and older [13]. This emphasizes once more the importance to distinguish UTIs in men as an own entity. Further, for the implementation of empirical therapy recommendations studies considering men only are crucial.

Despite the important role of *E. faecalis* in monomicrobial infections in men it is involved as co-infecting pathogen in nearly 50% of all polymicrobial infections. Experimental studies demonstrated the ability of *E. faecalis* to modulate the pathogenicity of gram-negative uropathogens [14, 15]. Unfortunately, by having no data on clinical outcomes we could not investigate any possible associations between the severeness of infections and *E. faecalis* as co-infecting pathogen.

Despite the high frequency of *E. faecalis* in men, data showing the causative character of this pathogen are sparse. In female studies the clinical relevance remains questionable as studies could demonstrate that E. faecalis can frequently be isolated from midstream urine but rarely from paired specimens of catheter urine [16]. In men such studies are missing and the role of *E. faecalis* in UTI remains unclear. Nevertheless, recent studies could reflect our findings of significantly higher frequencies of *E. faecalis* in UTI in men compared to women [7]. However, it is important to mention that the detection of E. faecalis in midstream urine can be caused by other infections such as prostatitis.

Recurrency is significantly more frequent in suspected UTI when *E. faecalis* is isolated compared to *E. coli.* The current European guideline recommends trimethoprim–sulfamethoxazole (TMP-SMX) as first line antibiotic for UTI in men, fluoroquinolones such as ciprofloxacin are first line choice as well, if the local resistance is below 10% [17]. Nevertheless, the activity of TMP-SMX is, as stated by the EUCAST, uncertain against *Enterococci* and clinical outcome isn’t predictable by AST [11]. This could indicate the use of inappropriate empirical treatment with antibiotics being inactive against *E. faecalis* but active against *E. coli.* In line*,* higher recurrency rates in UTI caused by other pathogens than *E. coli* are reflected in other studies as well [18].

Nevertheless, the recurrency rates not only differed between species but within species. Recurrency rates in suspected UTI when *E. faecalis* is isolated differed statistically significantly and stepwise between age groups, with men aged 18 to 29 years having the lowest recurrence rates with 12%. As causal for this result we discuss two important points. Firstly, the susceptibility of *E. faecalis* against ciprofloxacin is statistically significantly higher in men 18 to 29 years compared to older men. Secondly, ciprofloxacin is more commonly used in younger individuals due to its side-effects, such as tendon rupture, being more pronounced in the elderly [19]. Taken together, the ciprofloxacin usage is more, and ciprofloxacin resistance is less frequent in men 18 – 29 years compared to older individuals. As the german and european guideline recommend ciprofloxacin as one of the empirical first-line agents [3, 17] guideline based empirical antibiotic treatment eventually has a higher likelihood of treatment success in younger men. Further, our findings favor age dependent empirical therapy recommendations. Age related differences in resistance rates regarding quinolone resistance in *E. coli* isolates are reflected in female outpatient only studies as well [20]. Importantly, we excluded all resistance rates from 2020 in our study due to the unpredictable influence of the current Covid-19 pandemic.

In total the ciprofloxacin resistance in *Enterococci* is not changing over time. This is especially important as the ambulatory fluoroquinolone prescription declined in Germany from 101 per 1000 in 2010 to 60 per 1000 insured individuals [21]. This could on the one hand indicate a pronounced delay in AMR-changes in response to changes of prescribing behavior and on the other hand the necessity to further decrease quinolone prescription in the ambulatory setting.

As the nitrate-test is a measure of nitrate reducing bacteria (i.e. *enterobacteria*), the high incidence of *E. faecalis* in male patients highly questions its use in male patients due to the missing capability of *E. faecalis* to reduce nitrates. This eventually would lead into false negative results [22]. This conclusion is reflected in studies with elderly female patients where the nitrate test had a low negative predictive accuracy among other things due to higher frequencies of gram positive bacteria in the elderly [23].

In contrast, these findings again support the German guideline recommendations to always perform urine-cultures prior to empirical antibiotic use in men [3].

This study has several limits. Among other things its retrospective design inherently gives risk for bias. Next, we did not have any clinical data such as comorbidities, outcome or severeness of symptoms. Due to the lack of clinical data, we cannot rule out asymptomatic bacteriuria as diagnosis. Therefore, the high frequency of *E. faecalis* could illustrate other things but infection such as contamination or commensal. Nevertheless, in male patients screening for UTI is not recommended by German guideline [3]. That being said, it is unlikely that urine specimens obtained from asymptomatic bacteriuria in male patients are send to laboratories.

## Conclusions

*Enterococcus faecalis* is a highly relevant bacteria in male patients with young men being predominantly at risk. Negative nitrate-test results therefore cannot be recommended to exclude UTI. The high age-dependent ciprofloxacin resistance of *E. faecalis* supports the empirical use of ciprofloxacin in young adults. Frequent inappropriate empirical therapy indicated by high recurrency rates in UTI caused by *E. faecalis* emphasize the importance of pathogen identification and antimicrobial susceptibility testing in men suffering from UTI.

### Supplementary Information

**Additional file 1:** **Supplementary Table 1. **Frequency of pathogens detected in midstream specimens of urine in male outpatients stratified by age and polymicrobial infections, Germany, 2015 – 2020 (*n* = 120,961).

## Data Availability

The datasets generated and/or analysed during the current study are not publicly available due to privacy policies but are available from the corresponding author on reasonable request.

## Abbreviations

AMR: Antimicrobial resistance
CI: Confidence interval
CIP: Ciprofloxacin
ECUAST: European committee on antimicrobial susceptibility testing
MALDI-TOF: Matrix-assisted laser desorption/ionization - Time of flight
MIQ: Mikrobiologisch-infektiologische Qualitätsstandards
SD: Standard deviation
UTI: Urinary tract infection
