# Supplementary material for: High frequency of Enterococcus faecalis detected in urinary tract infections in male outpatients – a retrospective, multicenter analysis, Germany 2015 to 2020
Source: BMC Infect Dis. 2023 Nov 18;23:812. doi: 10.1186/s12879-023-08824-6 (PMC10657571; doi:10.1186/s12879-023-08824-6)
Supplement: Supplementary file 1 — Additional file 1: Supplementary Table 1. Frequency of pathogens detected in midstream specimens of urine in male outpatients stratified by age and polymicrobial infections, Germany, 2015 – 2020 (n = 120,961). [file 12879_2023_8824_MOESM1_ESM.docx]

| **Supplementary table 1:** Frequency of pathogens detected in midstream specimens of urine in male outpatients stratified by age and polymicrobial infections, Germany, 2015 – 2020 (n = 120,961) | | | | | | | | | | | | |
| --- | --- | --- | --- | --- | --- | --- | --- | --- | --- | --- | --- | --- |
|  | **Overall** (N = 120,961) | | | | **Monomicrobial** (N = 77,864) | | | | **Polymicrobial** (N = 43,097) | | | |
|  | Total | 18-29 yrs (N = 2,543) | 30-69 yrs (N = 47,277) | $\geq$ 70 yrs (N = 71,141) | Total | 18-29 yrs (N = 1,897) | 30-69 yrs  (N = 32,943) | $\geq$70 yrs  (N = 43,024) | Total | 18-29 yrs  (N = 646) | 30-69 yrs  N = 14334) | $\geq$ 70 yrs  (N = 28117) |
| **Pathogen** | | | | | | | | | | | | |
| *E. coli* | 48,439 (40.0%) | 1,010 (39.7%) | 22,072 (46.7%) | 25,357 (35.6%) | 37,596 (48.3%) | 816  (43.0%) | 18,121 (55.0%) | 18,659 (43.4%) | 10,843 (25.2%) | 194  (30.0%) | 3,951 (27.6%) | 6,698  (23.8%) |
| *E. faecalis* | 19,471 (16.1%) | 426  (16.8%) | 7,338 (15.5%) | 11,707 (16.5%) | 9,353 (12.0%) | 264  (13.9%) | 3,783 (11.5%) | 5,306  (12.3%) | 10,118 (23.5%) | 162  (25.1%) | 3,555 (24.8%) | 6,401  (22.8%) |
| *P. mirabilis* | 11,177 (9.2%) | 222  (8.7%) | 3,349  (7.1%) | 7,606  (10.7%) | 6,406  (8.2%) | 146  (7.7%) | 1,922  (5.8%) | 4,338  (10.1%) | 4,771 (11.1%) | 76  (11.8%) | 1,427 (10.0%) | 3,268  (11.6%) |
| *K. pneumoniae* | 9,162 (7.6%) | 183  (7.2%) | 3,479  (7.4%) | 5,500  (7.7%) | 5,675  (7.3%) | 124  (6.5%) | 2,305  (7.0%) | 3,246  (7.5%) | 3,487  (8.1%) | 59  (9.1%) | 1,174  (8.2%) | 2,254  (8.0%) |
| *P. aeruginosa* | 6,691 (5.5%) | 69  (2.7%) | 1,930  (4.1%) | 4,692  (6.6%) | 3,390  (4.4%) | 38  (2.0%) | 1,028  (3.1%) | 2,324  (5.4%) | 3,301  (7.7%) | 31  (4.8%) | 902  (6.3%) | 2,368  (8.4%) |
| *Klebsiella spp.* | 4,872 (4.0%) | 81  (3.2%) | 1,606  (3.4%) | 3,185  (4.5%) | 2,781  (3.6%) | 59  (3.1%) | 979  (3.0%) | 1,743  (4.1%) | 2,091  (4.9%) | 22  (3.4%) | 627  (4.4%) | 1,442  (5.1%) |
| *Citrobacter spp.* | 4,516 (3.7%) | 48  (1.9%) | 1,480  (3.1%) | 2,988  (4.2%) | 2,674  (3.4%) | 38  (2.0%) | 902  (2.7%) | 1,734  (4.0%) | 1,842  (4.3%) | 10  (1.5%) | 578  (4.0%) | 1,254  (4.5%) |
| *S. aureus* | 3,840 (3.2%) | 56  (2.2%) | 1,179  (2.5%) | 2,605  (3.7%) | 2,396  (3.1%) | 41  (2.2%) | 768  (2.3%) | 1,587  (3.7%) | 1,444  (3.4%) | 15  (2.3%) | 411  (2.9%) | 1,018  (3.6%) |
| *Enterobacter spp.* | 3,719 (3.1%) | 69  (2.7%) | 1,295  (2.7%) | 2,355  (3.3%) | 2,304  (3.0%) | 46  (2.4%) | 858  (2.6%) | 1,400  (3.3%) | 1,415  (3.3%) | 23  (3.6%) | 437  (3.0%) | 955  (3.4%) |
| *Morganella spp.* | 2,141 (1.8%) | 32  (1.3%) | 776  (1.6%) | 1,333  (1.9%) | 1,071  (1.4%) | 19  (1.0%) | 400  (1.2%) | 652  (1.5%) | 1,070  (2.5%) | 13  (2.0%) | 376  (2.6%) | 681  (2.4%) |
| *β-hem.-Strept.^1^* | 1,994 (1.6%) | 98  (3.9%) | 1,101  (2.3%) | 795  (1.1%) | 1,383  (1.8%) | 78  (4.1%) | 810  (2.5%) | 495  (1.2%) | 611  (1.4%) | 20  (3.1%) | 291  (2.0%) | 300  (1.1%) |
| *Others^2^* | 4,939 (4.1%) | 249  (9.8%) | 1,672  (3.5%) | 3,018  (4.2%) | 2,835  (3.6%) | 228  (12.0%) | 1,067  (3.2%) | 1,540  (3.6%) | 2,104  (4.9%) | 21  (3.3%) | 605  (4.2%) | 1,478  (5.3%) |
| ^1^ β-hemolytic Strept.: Streptococcus agalactiae and Streptococcus pyogenes  ^2^ Others: inter alia Enterococcus spp.; Proteus spp.; Providencia spp.; Aerococcus urinae; Aerococcus sanguinicola; Enterococcus faecium; Mycoplasma/Ureaplasma; Corynebacterium urealyticum; Staphylococcus saprophyticus | | | | | | | | | | | | |
